# Efficient Delivery of Plasmid DNA Using Cholesterol-Based Cationic Lipids Containing Polyamines and Ether Linkages

**DOI:** 10.3390/ijms15057293

**Published:** 2014-04-28

**Authors:** Bieong-Kil Kim, Young-Bae Seu, Yun-Ui Bae, Tae-Won Kwak, Hyungu Kang, Ik-Jae Moon, Guen-Bae Hwang, So-Young Park, Kyung-Oh Doh

**Affiliations:** 1 School of Life Sciences & Biotechnology, Kyungpook National University, 1370 Sangyeokdong, Bookgu, Daegu 702-701, Korea; E-Mails: kbk8516@hanmail.net (B.-K.K.); ybseu@knu.ac.kr (Y.-B.S.); micro00tw@nate.com (T.-W.K.); hymnhim1@gmail.com (H.K.); daekom@hanmail.net (G.-B.H.); 2 Department of Physiology, Yeungnam University College of Medicine, 317-1 Daemyungdong, Daegu 705-717, Korea; E-Mails: neerg@hanmail.net (Y.-U.B.); sypark@med.yu.ac.kr (S.-Y.P.); 3 WelGENE Inc., 71B-4L, Hightech Sector 2, Sungseo Industrial Park 3, Dalseogu, Daegu 704-230, Korea; E-Mail: moonikjae@clearlab.com.sg

**Keywords:** cholesterol, cationic lipids, cyanoethylation, transfection, gene delivery, macropinocytosis

## Abstract

Cationic liposomes are broadly used as non-viral vectors to deliver genetic materials that can be used to treat various diseases including cancer. To circumvent problems associated with cationic liposome-mediated delivery systems such as low transfection efficiency and serum-induced inhibition, cholesterol-based cationic lipids have been synthesized that resist the effects of serum. The introduction of an ether-type linkage and extension of the aminopropyl head group on the cholesterol backbone increased the transfection efficiency and DNA binding affinity compared to a carbamoyl-type linkage and a mono aminopropyl head group, respectively. Under optimal conditions, each liposome formulation showed higher transfection efficiency in AGS and Huh-7 cells than commercially available cationic liposomes, particularly in the presence of serum. The following molecular structures were found to have a positive effect on transfection properties: (i) extended aminopropyl head groups for a strong binding affinity to plasmid DNA; (ii) an ether linkage that favors electrostatic binding to plasmid DNA; and (iii) a cholesterol backbone for serum resistance.

## 1. Introduction

Successful transfer of genetic material including plasmids, antisense and decoy oligonucleotides, and siRNA is a significant issue in the field of gene therapy [1]. Typically, genetic material is easily degraded by nucleases and is ineffectively delivered into cells [2]. Consequently, the development of safe and effective vectors for delivery into cells is needed. Despite the higher efficiency of viral vectors, non-viral vectors have been actively investigated because of their ease of synthesis, low immune response, and safety [3–7]. Moreover, non-viral vectors can deliver diverse chemical agents and nucleic acids. Therefore, non-viral systems are more favorable in the field of cancer therapy, and several clinical trials have been performed using liposomes [7,8]. Of the available non-viral vectors, cationic lipids have been the most extensively studied. The structure of cationic lipids generally comprises a cationic head group attached to a hydrophobic domain by a linker [9].

The cationic centers of the head group are typically tertiary [10,11] and quaternary amines [12,13]. Tertiary amines with methyl and ethyl group are generally less basic than their primary amine counterparts. Therefore, primary amine head groups may possess stronger DNA binding activity than tertiary amines. Polycations, such as poly-l-lysine and poly-l-ornithine, contain primary amino groups [14,15] and bind DNA in a strong charged interaction. Ether linkage in a cholesterol backbone is more efficient than other linkage types for gene delivery [16,17]. Efficient gene delivery of quaternary ammonium cationic lipids by using a cholesterol backbone and an ether linkage has been reported previously [18–20]. The hydrophobic domains are generally long-chain fatty acids or a cholesterol derivative. Because cholesterol is less toxic than other cationic lipids [2], it has been used as the major lipid component of liposomes for the delivery of genes [21] and chemical agents [22,23].

For efficient delivery, stability in serum is a major concern, particularly *in vivo* [24]. The presence of serum in the cell culture medium decreases the degree of lipid/DNA complex (lipoplex) association with cells [25] and the amount of nucleic acid delivered to the nuclei [26]. The presence of serum also affects the intracellular processing of lipoplexes [27]. In cell culture experiments, cholesterol has been shown to confer resistance to transfection in serum [28]. Serum-enhanced siRNA delivery using cholesterol derivative-based liposomes has been reported [29], and the conjugation of cholesterol to the 3′ end of the siRNA sense strand by means of a pyrrolidine linker improves the *in vivo* pharmacological properties of siRNAs [30]. Therefore, cholesterol-based liposomes may be a more appropriate tool than a commercial cationic liposome such as Lipofectamine to extend *in vitro* studies *in vivo*.

In this study, we focused on the development and application of cationic liposome delivery systems that can increase serum stability and transfection efficiency based on our preliminary data [17]. The highly effective cholesterol derivatives containing polyamine heads and ether linkages were synthesized by aminopropyl chain extension using cyanoethlyation. We hypothesize that this derivative enhances the cellular delivery of plasmids through liposomes in the presence of serum.

## 2. Results

### 2.1. Cationic Lipids

**Figure 1.**
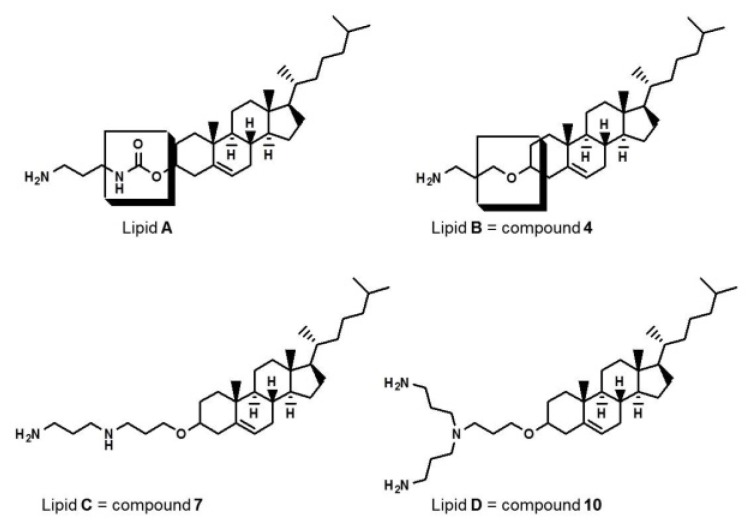
Structures of cholesterol-based cationic lipids. Lipid **A** has a carbamoyl-linked structure and lipids **B**–**D** have ether-linked structures.

Cholesterol-based cationic lipids with ether linkages were synthesized (Scheme S1) according to a method previously described [17]. To compare ether synthesized by cyanoethylation and carbamoyl linkages on the cholesterol backbone, we synthesized lipid **A** (Figure 1), which contained a carbamoyl-linked propylamine head that was easily synthesized from cholesteryl chloroformate, using the methods described by Tsutomu *et al.* [31]. Structures of all the synthetic intermediates and target lipids shown in Scheme S1 were confirmed by proton nuclear magnetic resonance (^1^H-NMR). Final compounds were analyzed using gas chromatography mass spectrometry (GC–MS) and fast atom bombardment mass spectrometry (FAB–MS) to confirm the identity of the molecular ions.

### 2.2. In Vitro Characterization of Transfection Efficiencies at Different Lipid:DOPE Weight Ratios

**Figure 2.**
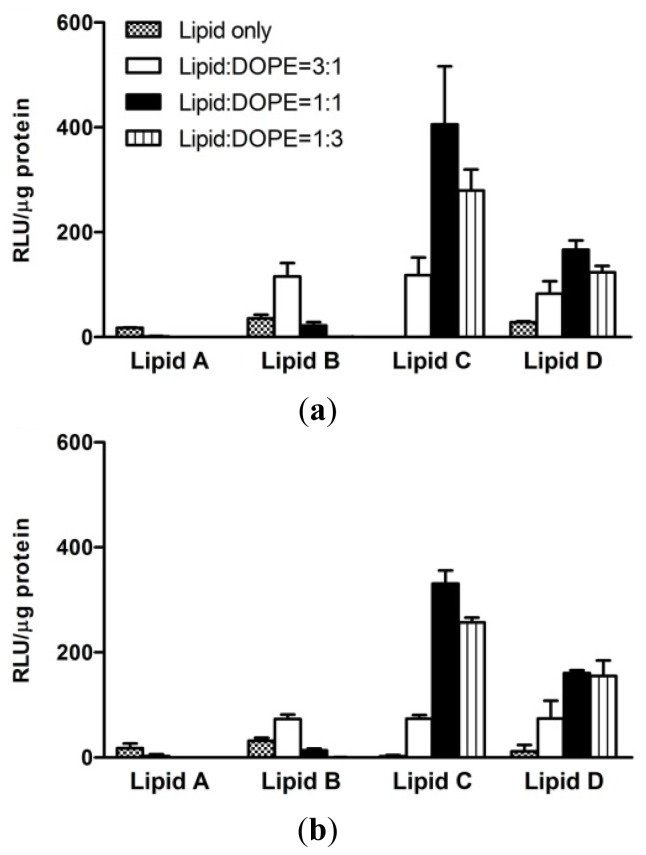
Transfection efficiencies at different lipid:DOPE weight ratios. *In vitro* transfection efficiencies of lipid/DNA complexes were measured using a luciferase assay at various lipid:DOPE weight ratios in AGS (**a**) and Huh-7 (**b**) cells. The lipid/DNA (N/P) weight ratio was kept constant at 2. Each bar represents the mean ± standard deviation (SD) of experiments performed in triplicate. RLU, relative luminescence units.

The addition of a neutral co-lipid such as 1,2-dioleoyl-*sn*-glycero-3-phosphoethanolamine (DOPE) to cationic liposomes reportedly enhances gene delivery [32]. Thus, we mixed synthesized cationic lipids with various ratios of DOPE. Figure 2 shows the transfection efficiencies of lipid/DNA complexes in AGS and Huh-7 cells in response to changes in lipid:DOPE weight ratios. The lipid/DNA (N/P) weight ratio was kept constant at a value of 2. The addition of DOPE to cholesterol derivatives increased luciferase activity, except in the case of lipid **A**. An optimal lipid:DOPE weight ratio was 3:1 for lipid **B** and 1:1 for lipids **C** and **D** in both cell types. The transfection efficiencies of lipids **A**–**C** did not increase when three times more DOPE was added than lipids (lipid:DOPE = 1:3). Only lipid **D** showed a marked increase in luciferase activity under these conditions. Lipids **A** and **B** had the same structure excluding the linkage. Ether linkage-containing lipid **B** showed increased transfection efficiency compared with carbamoyl-linked lipid **A**. On the other hand, lipids **B**–**D** that contained different head groups but the same ether linkage, showed different transfection efficiencies depending on the addition of DOPE.

### 2.3. In Vitro Transfection Efficiencies in Response to Changes in N/P Weight Ratio at an Optimized Lipid:DOPE Ratio

**Figure 3.**
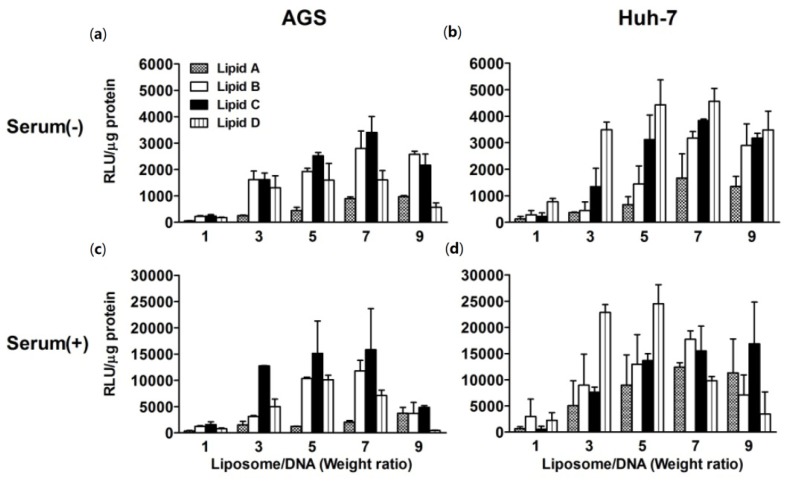
*In vitro* transfection efficiencies of liposomes **A**–**D** at different liposome:DNA weight ratios. *In vitro* transfection efficiencies of liposomes **A**–**D** in AGS (**a**,**c**) and Huh-7 (**b**,**d**) cells at optimized lipid:DOPE weight ratios (cationic lipids only for liposome **A**, 3:1 for liposome **B**, and 1:1 for liposome **C** and **D**) when using DOPE as a co-lipid. The incubations were performed in the presence (**c**,**d**) or absence (**a**,**b**) of serum. The *x*-axes show cationic liposome/DNA weight ratios while the concentration of DNA (0.2 μg/well) was kept constant. Bars represent the mean ± SD of experiments performed in triplicate.

A series of experiments were performed using an optimal lipid:DOPE ratio, as determined above, to further assess the transfection properties of liposomes. Transfection efficiencies at different N/P weight ratios in AGS and Huh-7 cells were measured using a luciferase assay (Figure 3). Transfection efficiencies of each liposome showed different results depending on the liposome/DNA weight ratio. The optimal conditions were different for AGS and Huh-7 cells, and the presence of serum caused marked changes in luciferase activity. In AGS cells, liposomes **B** and **C** were generally more potent in delivering DNA than liposomes **A** and **D** in the presence (Figure 3c) and absence (Figure 3a) of serum, regardless of the N/P weight ratios. In Huh-7 cells, however, liposome **D** induced higher luciferase activity than other liposomes in the absence of serum at all the N/P weight ratios tested (Figure 3b). Liposome **D** also induced higher luciferase activity than other liposomes in the presence of serum at weight ratios between 3 and 5 (Figure 3d).

### 2.4. Comparison with Commercial Liposomes

**Figure 4.**
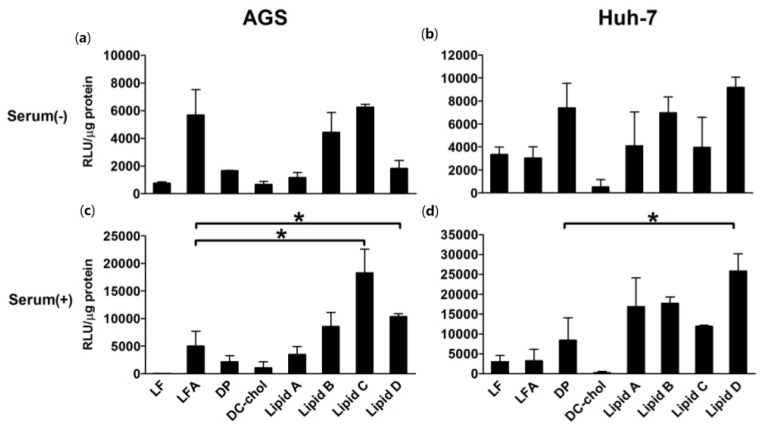
Transfection efficiencies compared to commercial liposomes. The transfection efficiencies of liposomes **A**–**D** were compared to that of commercial liposomes at optimal N/P weight ratios (5 for Lipofectin (LF) and Lipofectamine (LFA), and 7 for DOTAP/DOPE (DP) and DC–Chol) in AGS (**a**,**c**) and Huh-7 (**b**,**d**) cells by using a luciferase assay. The concentration of DNA (0.2 μg/well) was kept constant. The incubations were performed in the presence (**c**,**d**) and absence (**a**,**b**) of serum. Bars represent the mean ± SD of experiments performed in triplicate. * *p* < 0.05 *vs.* LFA (**A**,**C**) and DP (**B**,**D**).

**Figure 5.**
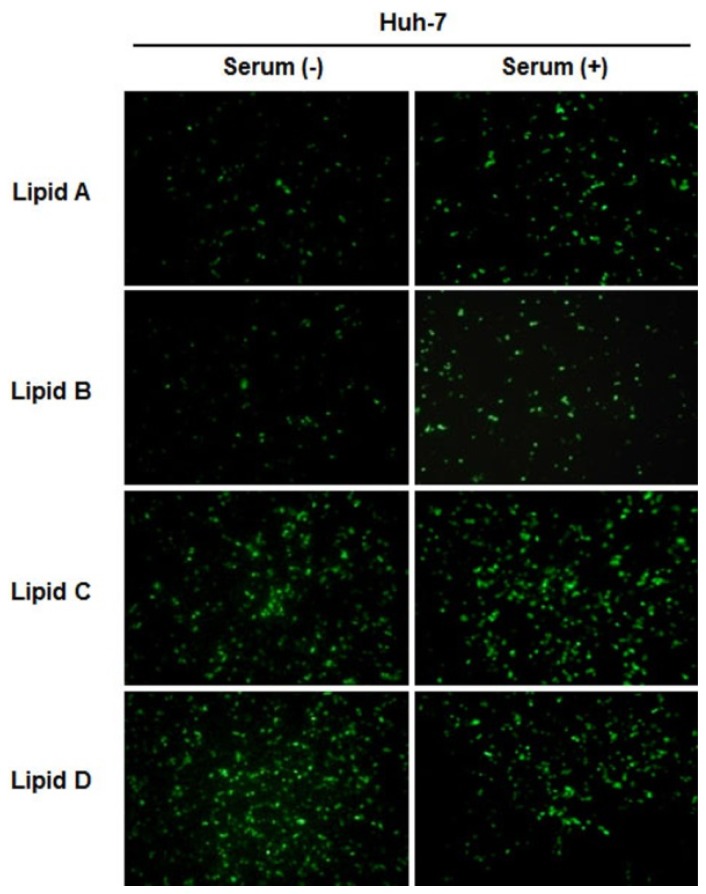
Expression of GFP delivered using liposomes **A**–**D**. The plasmid pCMVTnT-GFP (0.3 μg) was complexed with liposomes **A**–**D** and applied to Huh-7 cells. At the end of the transfection period, cells were examined under a fluorescent microscope.

We compared the transfection efficiencies of liposomes **A**–**D** that of commercial lipids such as Lipofectin, Lipofectamine, 1,2-dioleoyl-*sn*-glycero-3-trimethylammonium-propane (DOTAP)/DOPE, and 3β-[*N*,(*N*,*N*-dimethylaminoethane)-carbamoyl]cholesterol (DC–Chol) at the optimal N/P weight ratios for synthesized lipids and recommended ratios for commercial liposomes (Figure 4). Of the commercial liposomes, Lipofectamine showed the highest transfection efficiency in AGS cells in the absence of serum. Liposomes **B** and **C** showed similar luciferase activity compared to Lipofectamine in the absence of serum. Moreover, liposomes **B**–**D** induced in higher luciferase activity than Lipofectamine and liposome **C** had the highest activity of all the liposomes in the presence of serum in AGS. In Huh-7 cells, DOTAP/DOPE was the most effective of the commercial liposomes used in this experiment. Although the ability of liposomes **B** and **D** to deliver DNA into Huh-7 cells was comparable to that of DOTAP/DOPE in the absence of serum, liposomes **A**–**D** had a higher transfection activity than DOTAP/DOPE in the presence of serum. In both AGS and Huh-7 cells, the luciferase assays showed that the presence of serum increased the transfection efficiencies of liposomes **A**–**D** (Figures 3 and 4). The expression of GFP delivered using liposomes **A**–**D** was in accordance with the luciferase assay results (Figure 5).

### 2.5. In Vitro Cytotoxicity of Liposomes

**Figure 6.**
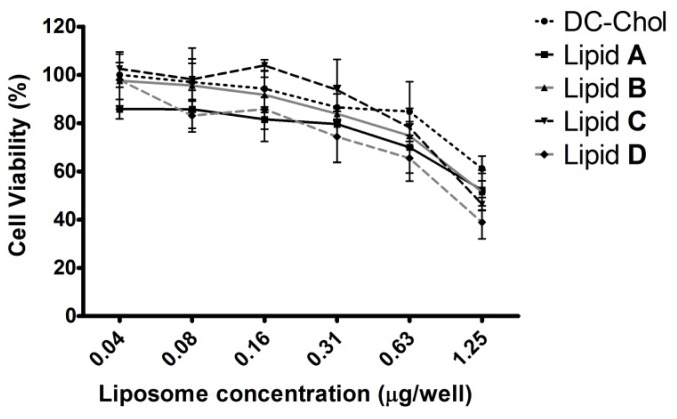
Cytotoxicity of liposomes **A**–**D** in AGS cells. AGS cells were treated with liposomes **A**–**D** and DC–Chol complexed with pcDNA–Luc plasmid. Cell viability was measured using a MTT assay at various concentrations of lipid/DNA complexes. Controls were treated with plasmid DNA only. The results are represented as the mean ± SD of experiments performed in triplicate.

Because cholesterol-based cationic lipids are less toxic than other cationic lipids [2], cholesterol has been used as the major lipid component in liposomes used for delivery of genes [21] and chemical agents [22,23]. We evaluated 3-(4,5-dimethylthiazol-2-yl)-2,5-diphenyltetrazolium bromide (MTT)-based cell viabilities of AGS cells exposed to liposomes **A**–**D** or DC–Chol at various lipid:DNA concentrations (Figure 6). Cell viabilities obtained in response to liposomes **A**–**D** did not significantly differ from those obtained after exposure to DC–Chol. Reasonable cytotoxicities at optimal concentrations for efficient transfection were observed.

### 2.6. Liposome/DNA Binding Interaction

**Figure 7.**
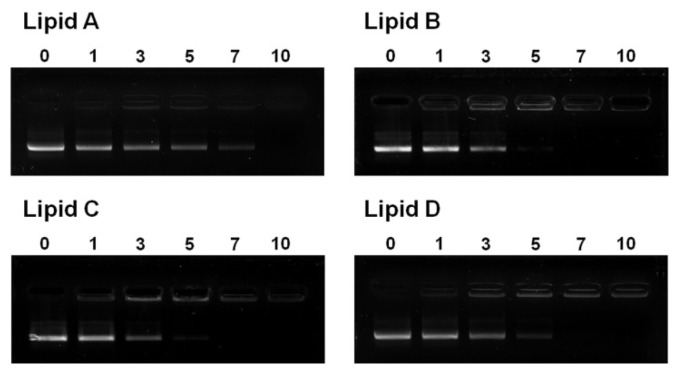
Gel retardation patterns of liposome/DNA complexes. Liposomes **A**–**D** were mixed with DNA at various weight ratios and then run though a 1% agarose gel. The mobility of the complexes was visualized using ethidium bromide staining. The N/P weight ratios are indicated at the top of each lane. Lanes marked “0” contained DNA alone and served as a control.

For cationic liposome- and polyamine-mediated transfections, electrostatic interaction between the DNA/carrier complex and the anionic plasma membrane is the first step [33]. To investigate the electrostatic binding interaction between DNA and the cationic liposomes, an electrophoretic gel retardation assay was performed. Representative electrophoretic gel patterns of liposome/DNA lipoplexes are shown in Figure 7. The liposomes were capable of completely inhibiting the electrophoretic mobility of plasmid DNA contained within lipoplexes prepared at N/P weight ratios higher than 10. At an N/P weight ratio of 7, free DNA migrated for liposome **A**, but not for liposomes **B**–**D**. Such gel patterns are consistent with the relatively weak liposome/DNA binding interactions of liposome **A**, which has the greatest amount of cationic lipids in the absence of the neutral lipid DOPE (cationic lipid/DOPE weight ratio is 3:1 for **B** and >1:1 for **C** and **D**). This result is consistent with the observation that the transfection efficiency of liposomes was the highest in luciferase assays at an N/P weight ratio of 7–10 for lipid **A** and 5–7 for lipids **B**–**D** (Figure 3). These data imply that liposomes are not sufficient to bind DNA at N/P weight ratios under 3 or 5. In addition, ratios over 7 or 10 are too high for the interaction with cell membranes.

### 2.7. Liposome Formulation in the Presence of Helper-Lipid DOPE in Aqueous Solution

To evaluate the effect of physicochemical properties on the transfection efficiency of liposomes **A**–**D**, the size and ζ-potential of liposomes **A**–**D** under optimal transfection conditions were measured. The particle size of the liposomes at optimum lipid **A**–**D**:DOPE weight ratios (Figure 2) ranged from 87–176 nm (Table 1). The liposome formed by lipid **A** in the absence of DOPE had the smallest particle size of the four liposomes. The remaining three liposomes had similar particle sizes. The positive surface charge of liposome vesicles varied between 42 and 59 mV, in the following order: liposome **A** < **B** < **C** < **D** (Table 1). Ether-linked amine heads (lipid **B**) had greater positive surface charge than carbamoyl-linked amine heads (lipid **A**). In addition, the positive surface charge of the liposomes increased as the number of cationic amines increased (lipid **B**–**D**). The presence of sufficient positive charge may facilitate its interaction with negatively charged DNA (Figure 7), allowing for the efficient delivery of plasmid DNA (Figure 4).

## 3. Discussion

We synthesized four cholesterol-based cationic lipids and optimized them for *in vitro* transfection. Cholesterol has been used for the formulation of liposomes used in DNA delivery. Several studies report that transfection efficiency is enhanced when cholesterol-derivatives are used [34–37]. The use of cholesterol in the formulation of lipoplexes has been shown to enhance transfection both *in vitro* and *in vivo* [28,38]. Furthermore, cholesterol-based liposomes showed resistance to serum inhibition [39,40] and possessed biocompatibility [2]. In general, cholesterol is effective in reducing the binding of serum proteins to lipoplexes [41], thereby improving transfection of DNA both *in vitro* and *in vivo* [42]. Our lipids also showed effective gene delivery and reasonably low cytotoxicity when used at an optimal concentration for transfection, especially in the presence of serum.

In the cationic lipids used for gene delivery, the head group was positively charged and bound to negatively charged DNA. Primary amine head groups have been shown to have superior transfection efficiency because of their strong positive charge [14,15]. Cationic lipids are often mixed with a neutral co-lipid such as DOPE [43,44]. The addition of DOPE to cationic liposomes reportedly enhances gene delivery [32] and contributes to enhanced bio-distribution to the target site *in vivo* [45]. Therefore, to clarify the effect of a positively charged head group on transfection, we compared three different head groups containing primary amines and observed different biological properties at optimal DOPE and N/P weight ratios. We found that the positive surface charge of liposomes increased with the number of cationic amines (lipid **B**–**D**). The presence of a sufficient positive charge may facilitate interaction with negatively charged DNA (Figure 7), stimulating efficient delivery of plasmid DNA (Figure 4). We used an ether linker between the amine head and hydrophobic cholesterol. Ether linkers in a cholesterol backbone are known to be more efficient for gene delivery than other linkage types [16,17]. Previously, we showed that alterations to the carbon length and electron-rich π-bond in the spacer between the amine head and cholesterol backbone have profound effects on transfection efficiency [18]. We found that ether-linked lipids were more effective in the delivery of genes compared to carbamoyl-linked lipids with the same head group (lipids **A** and **B**). We attribute this to the presence of more unshared electron pairs in carbamoyl linkers than in ether linkers. This may inhibit tight electrostatic binding to plasmid DNA and, consequently, more cationic liposomes would be required to completely binding them (Figure 7).

The particle size of liposome **A** was smaller than that of the other liposomes, possibly contributing to its low transfection efficiency. However, the size and ζ-potential of liposomes **B**–**D** were similar. The similar transfection efficiencies of these liposomes under optimal conditions can be attributed to their similar physicochemical properties. Although we evaluated the size and ζ-potential of liposomes **A**–**D** under optimal conditions, the presence of serum may change these properties. Typically, the presence of serum decreases transfection efficiency and intracellular gene expression [25]. However, cholesterol has been shown to resist these effects of serum [28]. Currently, we do not have a concrete explanation for serum-enhanced delivery of cholesterol-based liposomes; however, similar results were observed in a recent report that used siRNA [29]. The interaction between cholesterol and serum albumin was proposed to enhance intracellular delivery through low-density lipoprotein receptors [46]. The binding of proteins from sera or bodily fluids to nanoparticles in a phenomenon called “protein corona,” has been recently suggested as a factor that affects the interaction between nanoparticles and cell membranes [47–49]. Protein corona-modified lipoplexes may interact differently with cell membranes, potentially explaining the enhanced transfection efficiency of cholesterol-based lipids in the presence of serum. However, the manner in which cholesterol positively interacts with proteins has yet to be elucidated.

Several mechanisms of endocytosis are known to be involved in DNA delivery [50]. Macropinocytosis generates large endocytic vesicles of irregular size and shape through actin-driven invagination of the plasma membrane [51]. It may be relevant to protein corona-modified lipoplexes. Recent studies showed that macropinocytosis is the main route for lipoplex uptake [52,53]. Interestingly, we found that internalized liposome **B** colocalized with a macropinocytosis marker (Figure S1). Although we previously demonstrated that the clathrin-mediated pathway is the primary endocytic route for cholesterol-based lipoplexes [54], we observed that macropinocytosis also has a role. Although the efficiency of a transfection system *in vitro* is not always an indicator of its success *in vivo*, *in vitro* transfection screens can rapidly and reproducibly generate preliminary information that can be used to modify liposome/DNA formulations and improve their stability.

## 4. Experimental Section

### 4.1. General Procedures and Materials

Gas chromatography mass spectrometry (GC-MS) data were acquired with a HP 6890 and Agilent 5973N MSD instrument (Agilent, Santa Clara, CA, USA). Fast atom bombardment mass spectrometry (FAB-MS) data was acquired with a high resolution mass spectrometer (JEOL JMS-700, Tokyo, Japan). ^1^H-NMR spectra were recorded on a Varian 300 MHz FT-NMR spectrometer. Cholesterol, reagents and organic solvent were purchased from Aldrich and used without further purification. The progress of the reactions was monitored by thin-layer chromatography on silica gel plates from Merck (1.05554; Darmstadt, Germany). Column chromatography was performed with silica gel (70–230 mesh, Merck). Lipofectamine and Lipofectin were purchased from Invitrogen (Carlsbad, CA, USA). DOTAP was purchased from Roche Molecular (Penzberg, Germany). DC–Chol was purchased from Sigma (St. Louis, MO, USA). DOPE was purchased from Fluka (Buchs, Switzerland). Cell culture media, Opti-MEM and fetal bovine serum (FBS) were purchased from Gibco (Carlsbad, CA, USA). AGS (human stomach cancer), Huh-7 (human hepatocarcinoma) and COS-7 (african green monkey kidney) cell lines were procured from the Korean Cell Line Bank. Cells were grown at 37 °C in RPMI 1640 or Dulbecco’s Modified Eagle’s Medium (DMEM) with 10% FBS in a humidified atmosphere containing 5% CO_2_/95% air. Synthesis of cationic lipids and its spectral data were described in supporting information.

### 4.2. Liposomes

#### 4.2.1. Preparation of Liposomes

For preparation of liposomes, the cationic lipids and DOPE were mixed in anhydrous organic solvent (a mixture of CHCl_3_ and MeOH) at an appropriate ratio, and the solvent was evaporated under vacuum. The film of cationic lipid and DOPE was then resuspended in sterile water to give total lipid concentrations of 1 mg/mL for *in vitro* transfection. The mixture was subsequently sonicated for 20 min at 40 °C to form the liposomes, and passed through a Mini-Extruder (Avanti Polar Lipids, Alabaster, AL, USA) equipped with double-layered 200 nm polycarbonate membrane filters 10 times. The obtained liposomes were stored at 4 °C prior to use. For comparison with lipids **A**–**D**, DOTAP, DC–Chol, Lipofectin and Lipofectamine were purchased.

#### 4.2.2. Size Measurement and Zeta Potential Analysis of Liposomes

The particle size distribution and zeta potential of a liposome’s dispersion were determined at 25 °C by the dynamic light scattering method on an ELS-Z (Otsuka Electronics Co., Osaka, Japan). The concentration of a liposome was 100 μg in 3 mL of Milli Q water. The particle size was measured three times in a set of fifty repetitions, and zeta potential was measured three times. Data were analyzed using a software package (ELS-Z software, Otsuka Electronics Co., Osaka, Japan) supplied by the manufacturer.

### 4.3. Lipoplexes

#### 4.3.1. Preparation of Plasmids DNA

The reporter plasmid pcDNA–Luc used for *in vitro* transfection was a plasmid of 5.149 kb containing the firefly luciferase reporter gene sequence. The plasmid coding for Green Fluorescent Protein (pCMVTnT-GFP) was purchased from Welgene (Daegu, Korea). DNA plasmids were amplified in the *Escherichia coli* XL 1-Blue strain and purified by using a Qiagen maxi-kit (Qiagen Inc., Hilden, Germany) according to the manufacturer’s instructions. DNA purity was determined by agarose gel electrophoresis and by measuring optical density (OD). DNA having OD_260_/OD_280_ ≥1.8 was used in this study.

#### 4.3.2. Preparation of Lipoplexes

A liposome solution was separately prepared by diluting an appropriate amount of the initial liposomal stock solution with Opti-MEM to reach a final volume of 50 μL. To this liposome solution, 50 μL of the DNA stock solution was added, and (after mixing) the tube was incubated for 10 min at room temperature. The content was then diluted with 100 μL of Opti-MEM (final volume of the lipoplex stock solution was 200 μL).

#### 4.3.3. DNA-Binding Assay

The DNA-binding ability of the cationic liposomes was assessed by a gel retardation assay on a 1% agarose gel (prestained with ethidium bromide) across the varying lipid/DNA weight ratios. In a total volume of 60 μL, pCMVTnT-GFP (1 μg) was mixed with varying amounts of cationic lipids and the mixture was incubated at room temperature for 10 min. Loading buffer (7 μL, 6×) (0.25% bromophenol blue in 40% (*w*/*v*) sucrose in H_2_O) was added to the samples, and the resulting solution (35 μL) was loaded on each well. The samples were run at 200 mV for 20 min, and the DNA bands were visualized in the gel documentation unit (Alpha Innotech, San Leandro, CA, USA).

### 4.4. Cell Biology

#### 4.4.1. *In Vitro* Transfection

The lipoplexes were tested for their ability to transfer DNA in AGS, Huh-7 and COS-7 cells. The cells were maintained in 10% FBS enriched medium at 37 °C in a humidified atmosphere of 5% CO_2_/95% air. The following media were used: RPMI 1640 for AGS, DMEM for Huh-7 and COS-7 cell line. Twenty-four hours prior to transfection, the cells were transferred to 48-well Falcon plates at a density of 40,000 cells per well. Each well received 200 μL of appropriate medium, and the plate was incubated in the same conditions as above. Immediately before transfection, the medium was removed and the cells from each well were briefly washed with 200 μL of sterile phosphate buffered saline (PBS). After removal of the PBS solution, each well received 200 μL of lipoplex stock solution, and the plate was returned to the incubator for 4 h. An additional 200 μL of medium (20% FBS) was added to each well, and the plate was incubated for a further 24 h, after which the luciferase and protein contents were assessed. In the case of presence of serum (serum+), each well received 200 μL of medium (20% FBS) and then 200 μL of lipoplex stock solution was added to each well. For control transfection, commercial liposomes were tested in order to reveal the best conditions within the range of the manufacturer’s protocol, and used in Figure 4 in those optimal conditions.

#### 4.4.2. Luciferase Assay

Twenty-four hours after transfection, the medium was removed and the wells were washed briefly with 200 μL of PBS. After removal of PBS, the cells were collected by adding 100 μL of 1× reporter lysis buffer (Promega, Fitchburg, WI, USA) to each well and the cell lysates were used for luciferase and protein assays. For the luciferase assay, 20 μL of cell lysate was transferred to a test tube and assessed directly by means of a Lumat LB 9507 luminometer (Berthold Detection Systems, Pforzheim, Germany), using a luciferase assay kit from Promega. The protein content was quantified using a bicinchoninic acid (BCA) assay (PIERCE, Rockford, IL, USA). The BCA assay was prepared as specified by the manufacturer. Forty microliters of cell lysate was mixed with 1 mL of BCA reagent in an acrylic cuvette, and the solution was incubated for 1 h at 37 °C. The light absorption of the solution was then read at 562 nm by means of a Beckman DU-600 UV-vis spectrophotometer (Palo Alto, CA, USA), and the protein content was estimated by comparison to bovine serum albumin standards. The luciferase activity was normalized by the protein content and expressed as relative luminescence units/μg of protein (RLU/μg protein).

#### 4.4.3. Fluorescence Microscopy: GFP Expression

The day before transfection, Huh-7 cells (4 × 10^4^ per well) were seeded into a 48-well plate. The plasmids pCMVTnT-GFP (0.3 μg) complexed with liposomes at various weight ratios, were added to the cells, according to the transfection protocol described above. After 24 h of incubation, the medium was removed, and the cells were rinsed twice with PBS. Fluorescence expression was observed with a Leica DM-IRE2 fluorescence microscope (Solms, Germany).

#### 4.4.4. Cytotoxicity Assay

AGS cells (1 × 10^4^ per well) were seeded into a 96-well plate. The following day, the culture medium was replaced with 100 μL of plasmid pcDNA–Luc only as untreated control or 100 μL of lipid/DNA complexes at various concentrations in Opti-MEM medium. After 4 h, additional 100 μL of medium (20% FBS) was added to each well, and the plate was incubated for a further 24 h. The medium was changed with 80 μL of fresh medium and 20 μL (100 μg) of MTT solution at 5 mg/mL was added to the cells for an additional 4 h. The formazan crystals were dissolved in 200 μL of dimethylsulfoxide. Absorbance was measured at 570 nm on a VERSAmax microplate reader (Molecular Devices, Sunnyvale, CA, USA) and used to calculate the percentage of viable cells compared to untreated cells. Results were expressed as percent viability = [(A_treated cells_ − A_background_)/(A_untreated cells_ − A_background_)] × 100.

#### 4.4.5. Statistical Analysis

The statistical significance of the difference between groups was evaluated by one-way ANOVA and Tukey’s *post hoc* test. Asterisks indicate statistically significant differences (*p* < 0.05).

## 5. Conclusions

In summary, this report describes the synthesis, characterization and transfection biology of cholesterol-based cationic lipids containing a stable ether bond in their spacer. Under optimal conditions, each liposome formulation showed higher transfection efficiency in AGS and Huh-7 cells than commercially available cationic liposomes, particularly in the presence of serum with reasonable cytotoxicity. The following molecular structures were found to have a positive effect on transfection properties: (i) extended aminopropyl head groups for a strong binding affinity to plasmid DNA; (ii) an ether linkage that favors electrostatic binding to plasmid DNA; and (iii) a cholesterol backbone for serum resistance.

## Supplementary Information

### Synthesis of Cationic Lipids

#### Synthesis of 3β-cholest-5-en-3-yl *N*-(3-aminopropyl) carbamate, lipid A

Lipid **A** was synthesized according to the method of Tsutomu *et al*. [31]. Its spectral data was the same as that reported in the literature.

#### Synthesis of 2-cyanoethyl-*O*-β-cholesterol ether 2

Acrylonitrile (13.3 g, 251 mmol) was added drop-wise to a mixture of aqueous KOH (*w*/*w* 40%) 10 mL, 1,4,7,10,13,16-hexaoxacyclooctadecane (18-crown-6) (1.32 g, 5 mmol) and cholesterol **1** (19.3 g, 50 mmol) dissolved in 300 mL of CH_2_Cl_2._ This mixture was stirred at room temperature overnight. The solvent was removed on a rotary evaporator. The residue was then taken in 300 mL of hexane, washed sequentially with water (2 × 100 mL) and 100 mL a salt saturated solution, dried over anhydrous magnesium sulfate, and filtered. Hexane was removed from the filtrate on a rotary evaporator. Silica gel column chromatographic purification of the resulting residue using chloroform only as the eluent afforded 22.0 g of pure 2-cyanoethyl-*O*-β-cholesterol ether **2** (100% yield): ^1^H-NMR (300 MHz, CDCl_3_) δ (ppm): 0.68 (s, 3 H; H-18′), 0.86–0.87 (d, *J* = 6.6 Hz, 6 H; H-26′, H-27′), 0.91 (d, *J* = 6.6 Hz, 3 H; H-21′), 1.00–2.05 (m, 29 H; cholesterol), 2.16–2.39 (m, 2 H; H-24′), 2.58 (t, *J* = 6.3 Hz, 2 H; H-2), 3.22 (m, 1 H; H-3′), 3.70 (t, *J* = 6.3 Hz, 2 H; H-1), 5.36 (d, *J* = 5.4 Hz, 1 H; H-6′).

#### General Method for the Synthesis of Intermediates (Compound 3, 6, and 9)

Appropriate 2-cyanoethyl ether derivative was added in dry methanol (30 mL) to a round flask with 2 equiv of NiCl_2_·6H_2_O, and then 3–5 equiv of crystalline Boc-ON (1.26 g, 5.76 mmol) were added. After stirring for 10 min, 10–15 equiv of NaBH_4_ was added in small portions over 30 min. The reaction mixture was allowed to stir at room temperature for 2–3 h. The black precipitate was removed by filtration, and hexane was added to extract the product. The hexane phase was separated, and the methanol phase washed repeatedly with hexane. The combined hexane phase was dried over anhydrous magnesium sulfate, and filtered. Hexane was removed from the filtrate on a rotary evaporator. Silica gel column chromatographic purification of the resulting residue, using ethylacetate in hexane as the eluent, afforded a pure-white solid compound in 68%–93% yield.

Compound **3**, 3-carboxyaminopropyl-*O*-β-cholesterol ether (93% yield). ^1^H-NMR (300 MHz, CDCl_3_) δ (ppm): 0.65 (s, 3 H; H-18′), 0.84 (d, *J* = 6.6 Hz, 6 H; H-26′, H-27′), 0.89 (d, *J* = 6.6 Hz, 3 H; H-21′), 0.98–1.60 (m, 24 H; cholesterol), 1.42 (s, 9 H; H-Boc), 1.70–2.00 (m, 7 H; H-2, H-2′, H-7′, H-8′), 2.10–2.37 (m, 2 H; H-24′), 3.07–3.22 (br m, 3 H; H-3, H-3′), 3.51 (t, *J* = 5.7 Hz, 2 H; H-1), 4.89 (br s, 1 H; NH), 5.32 (d, *J* = 5.1 Hz, 1 H; H-6′).

Compound **6**, 3-carboxyaminopropyl-3-carboxyaminopropyl-*O*-β-cholesterol ether (80% yield). ^1^H-NMR (300 MHz, CDCl_3_) δ (ppm): 0.65 (s, 3 H; H-18′), 0.84 (d, *J* = 6.6 Hz, 6 H; H-26′, H-27′), 0.89 (d, *J* = 6.6 Hz, 3 H; H-21′), 0.97–1.60 (m, 24 H; cholesterol), 1.42 (s, 9 H; H-Boc), 1.44 (s, 9 H; H-Boc), 1.60–2.20 (m, 9 H; H-2, H-2′, H-5, H-7′, H-8′), 2.11–2.35 (m, 2 H; H-24′), 3.07–3.25 (m, 7 H; H-3, H-3′, H-4, H-6), 3.43 (t, *J* = 6.3 Hz, 2 H; H-1), 5.31 (d, *J* = 5.1 Hz, 1 H; H-6′), GC-MS (EI) *m*/*z* = 701.

Compound **9**, di-3-carboxyaminopropyl-3-aminopropyl-*O*-β-cholesterol ether (68% yield). ^1^H-NMR (300 MHz, CDCl_3_) δ (ppm): 0.66 (s, 3 H; H-18′), 0.84–0.85 (d, *J* = 6.6 Hz, 6 H; H-26′, H-27′), 0.89 (d, *J* = 6.6 Hz, 3 H; H-21′), 0.98–1.56 (m, 24 H; cholesterol), 1.42 (s, 18 H; H-Boc), 1.60–2.00 (m, 11 H; H-2, H-2′, H-5, H-7′, H-8, H-8′), 2.10–2.40 (m, 2 H; H-24′), 2.40–2.44 (br m, 6 H; H-3, H-6, H-9), 3.05–3.20 (m, 5 H; H-3′, H-4, H-7), 3.46 (t, *J* = 6.3 Hz, 2 H; H-1), 5.25 (br s, 2 H; NH), 5.32 (d, *J* = 5.1 Hz, 1 H; H-6′), GC-MS (EI) *m*/*z* = 758.

#### General Method for the Synthesis of Target Lipids (Compound 4, 7, and 10)

The excess of trifluoroacetic acid was added drop-wise to a solution of appropriate target lipids protected by Boc in CH_2_Cl_2_. The reaction mixture was allowed to stir for 2–4 h at room temperature and then the excess trifluoroacetic acid was removed *in vacuo*. Silica gel column chromatographic purification of the resulting residue, using methanol in chloroform as the eluent, afforded a pure-white solid compound in 65%–85% yield.

Compound **4**, 3-aminopropyl-*O*-β-cholesterol ether (85% yield). ^1^H-NMR (300 MHz, CDCl_3_) δ (ppm): 0.65 (s, 3 H; H-18′), 0.84 (d, *J* = 6.6 Hz, 6 H; H-26′, H-27′), 0.89 (d, *J* = 6.6 Hz, 3 H; H-21′), 0.98–1.60 (m, 24 H; cholesterol), 1.77–2.00 (m, 7 H; H-2, H-2′, H-7′, H-8′), 2.10–2.37 (m, 2 H; H-24′), 3.07–3.22 (br m, 3 H; H-3, H-3′), 3.63 (t, 2 H; H-1), 5.32 (d, *J* = 5.1 Hz, 1 H; H-6′), 8.24 (br s, 3 H; NH_3_), GC-MS (EI) *m*/*z* = 443.

Compound **7**, 3-aminopropyl-3-aminopropyl-*O*-β-cholesterol ether (65% yield). ^1^H-NMR (300 MHz, CDCl_3_:CD_3_OD = 1:1) δ (ppm): 0.66 (s, 3 H; H-18′), 0.83 (d, *J* = 6.6 Hz, 6 H; H-26′, H-27′), 0.89 (d, *J* = 6.6 Hz, 3 H; H-21′), 0.97–1.56 (m, 24 H; cholesterol), 1.75–2.10 (m, 9 H; H-2, H-2′, H-5, H-7′, H-8′), 2.10–2.35 (m, 2 H; H-24′), 2.96–3.20 (m, 7 H; H-3, H-3′, H-4, H-6), 3.57 (t, *J* = 5.7 Hz, 2 H; H-1), 5.31 (d, *J* = 5.1 Hz, 1 H; H-6′), FAB-MS *m*/*z* = 501.4782.

Compound **10**, 3-aminopropyl-3-aminopropyl-*O*-β-cholesterol ether (67% yield). ^1^H-NMR (300 MHz, CDCl_3_:CD_3_OD = 1:1) δ (ppm): 0.70 (s, 3 H; H-18′), 0.87 (d, *J* = 6.6 Hz, 6 H; H-26′, H-27′), 0.93 (d, *J* = 6.3 Hz, 3 H; H-21′), 1.01–1.60 (m, 24 H; cholesterol), 1.80–2.40 (m, 13 H; H-2, H-2′, H-5, H-7′, H-8, H-8′, H-24′), 3.14–3.62 (br m, 13 H; H-1, H-3, H-3′, H-4, H-6, H-7, H-9), 5.36 (br d, 1 H; H-6′), GC-MS (EI) *m*/*z* = 558.

#### Synthesis of 2-cyanoethyl-3-aminopropyl-*O*-β-cholesterol ether 5 and di-2-cyanoethyl-3-aminopropyl-O-β-cholesterol ether 8

Acrylonitrile (1.31 g, 24.7 mmol) was added to a solution of 3-aminopropyl-*O*-β-cholesterol ether **4** (2.19 g, 4.94 mmol) in MeOH (10 mL). The reaction mixture was refluxed for 12 h, and then the solvent was removed on a rotary evaporator. Silica gel column chromatographic purification of the resulting residue, using 10% MeOH in CHCl_3_ as the eluent, afforded 1.03 g of pure 2-cyanoethyl-3-aminopropyl-*O*-β-cholesterol ether **5** (42% yield). Compound **8** that was not separated by the first column chromatography purification was retained with reaction residues. Therefore, to purify compound **8**, column chromatography was performed once more. Purification of the resulting residue on a silica gel column, with ethylacetate/hexane = 5:1 as the eluent, resulted in 1.39 g of pure di-2-cyanoethyl-3-aminopropyl-*O*-β-cholesterol ether **8** (51% yield; compound **5**, 42% yield; overall, 93% yield).

Compound **5**, 2-cyanoethyl-3-aminopropyl-*O*-β-cholesterol ether (42% yield). ^1^H-NMR (300 MHz, CDCl_3_) δ (ppm): 0.68 (s, 3 H; H-18′), 0.86–0.87 (d, *J* = 6.6 Hz, 6 H; H-26′, H-27′), 0.91 (d, *J* = 6.6 Hz, 3 H; H-21′), 1.00–1.60 (m, 24 H; cholesterol), 1.70–2.00 (m, 7 H; H-2, H-2′, H-7′, H-8′), 2.10–2.40 (m, 2 H; H-24′), 2.53 (t, *J* = 6.6 Hz, 2 H; H-5), 2.75 (t, *J* = 6.6 Hz, 2 H; H-3), 2.94 (t, *J* = 6.6 Hz, 2 H; H-4), 3.13 (m, 1 H; H-3′), 3.55 (t, *J* = 6.3 Hz, 2 H; H-1), 5.34 (d, *J* = 5.1 Hz, 1 H; H-6′).

Compound **8**, di-2-cyanoethyl-3-aminopropyl-*O*-β-cholesterol ether (51% yield). ^1^H-NMR (300 MHz, CDCl_3_) δ (ppm): 0.65 (s, 3 H; H-18′), 0.84 (d, *J* = 6.6 Hz, 6 H; H-26′, H-27′), 0.89 (d, *J* = 6.6 Hz, 3 H; H-21′), 0.98–1.60 (m, 24 H; cholesterol), 1.62–2.00 (m, 7 H; H-2, H-2′, H-7′, H-8′), 2.10–2.37 (m, 2 H; H-24′), 2.46 (t, *J* = 6.9 Hz, 4 H; H-5, H-8), 2.62 (t, *J* = 6.9 Hz, 2 H; H-3), 2.83 (t, *J* = 6.9 Hz, 4 H; H-4, H-7), 3.12 (m, 1 H; H-3′), 3.51 (t, *J* = 6 Hz, 2 H; H-1), 5.33 (d, *J* = 5.1 Hz, 1 H; H-6′).

COS-7 cells were seeded onto 12 mm coverslips at a density of 6 × 104 cells in 24-well plates for 24 h. The plasmid was covalently labeled with the Label IT Cy3 nucleic acid labeling kit (Mirus, Chicago, IL, USA). Oregon Green 488 dextran; 70,000 MW (50 μg/400 μL, Molecular probe, Leiden, The Netherlands) and Cy3-labeled DNA using liposome B were added to the cells for 1 h to allow simultaneous uptake in the presence of serum. Cells were fixed with 500 μL of 4% paraformaldehyde for 10 min at room temperature. Fluorescence images were taken via confocal microscopy (Leica TCS SP2, Solms, Germany) in sequential mode to eliminate emission cross-talk between two dyes.

Scheme S1. Synthesis of new cationic lipids. (**a**) Acrylonitrile, 18-crown-6, aqueous KOH/CH_2_Cl_2_ (yield: 100%); (**b**) NiCl_2_·6H_2_O, Boc_2_O, NaBH_4_/MeOH (yield: **3**, 93%; **6**, 80%; **9**, 68%); (**c**) TFA/CH_2_Cl_2_ (yield: **4**, 85%; **7**, 65%; **10**, 67%); (**d**) Acrylonitrile/MeOH, reflux (yield: **5**, 42%; **8**, 51%; overall, 93%).

Figure S1. Cellular uptake of the liposome B/DNA complex. We examined the colocalization of lipoplexes with Oregon Green 488-labeled dextran 70,000 molecular weight (dextran 70,000), a known macropinocytic pathway marker. COS-7 cells were incubated with dextran 70,000 (**green**) and lipoplex B containing Cy3-labeled DNA (**red**). Images were obtained using confocal microscopy 1 h after transfection. Scale bar: 10 μm.

## Figures and Tables

**Table 1.** Size and ζ-potential of liposomes.

Liposome composition	Size (nm)	ζ-potential (mV)
Lipid **A** only	87 ± 5	42 ± 8
Lipid **B**/DOPE = 3/1	176 ± 9	50 ± 5
Lipid **C**/DOPE = 1/1	142 ± 1	55 ± 1
Lipid **D**/DOPE = 1/1	162 ± 7	59 ± 1

## References

[1] El-Aneed A. (2004). An overview of current delivery systems in cancer gene therapy. J. Control. Release.

[2] Lv H., Zhang S., Wang B., Cui S., Yan J. (2006). Toxicity of cationic lipids and cationic polymers in gene delivery. J. Control. Release.

[3] Bajaj A., Kondiah P., Bhattacharya S. (2007). Design, synthesis, and *in vitro* gene delivery efficacies of novel cholesterol-based gemini cationic lipids and their serum compatibility: A structure-activity investigation. J. Med. Chem.

[4] Zhang S., Zhao B., Jiang H., Wang B., Ma B. (2007). Cationic lipids and polymers mediated vectors for delivery of sirna. J. Control. Release.

[5] Ewert K., Ahmad A., Evans H.M., Schmidt H.W., Safinya C.R. (2002). Efficient synthesis and cell-transfection properties of a new multivalent cationic lipid for nonviral gene delivery. J. Med. Chem.

[6] Wang L., MacDonald R.C. (2004). New strategy for transfection: Mixtures of medium-chain and long-chain cationic lipids synergistically enhance transfection. Gene Ther.

[7] Kaneda Y., Tabata Y. (2006). Non-viral vectors for cancer therapy. Cancer Sci.

[8] Siehl J.M., Thiel E., Schmittel A., Hutter G., Deckert P.M., Szelenyi H., Keilholz U. (2005). Ifosfamide/liposomal daunorubicin is a well tolerated and active first-line chemotherapy regimen in advanced soft tissue sarcoma: Results of a phase ii study. Cancer.

[9] Gao H., Hui K.M. (2001). Synthesis of a novel series of cationic lipids that can act as efficient gene delivery vehicles through systematic heterocyclic substitution of cholesterol derivatives. Gene Ther.

[10] Gao X., Huang L. (1991). A novel cationic liposome reagent for efficient transfection of mammalian cells. Biochem. Biophys. Res. Commun.

[11] Takeuchi K., Ishihara M., Kawaura C., Noji M., Furuno T., Nakanishi M. (1996). Effect of zeta potential of cationic liposomes containing cationic cholesterol derivatives on gene transfection. FEBS Lett.

[12] Kisoon N., Ariatti M., Moodley T. (2002). A novel cationic cholesterol derivative, its formulation into liposomes, and the efficient transfection of the transformed human cell lines hepg2 and hela. Drug Deliv.

[13] Reynier P., Briane D., Coudert R., Fadda G., Bouchemal N., Bissieres P., Taillandier E., Cao A. (2004). Modifications in the head group and in the spacer of cholesterol-based cationic lipids promote transfection in melanoma b16-f10 cells and tumours. J. Drug Target.

[14] Wu G.Y., Wu C.H. (1988). Receptor-mediated gene delivery and expression *in vivo*. J. Biol. Chem..

[15] Dong Y.H., Skoultchi A.I., Pollard J.W. (1993). Efficient DNA transfection of quiescent mammalian-cells using poly-l-ornithine. Nucleic Acids Res.

[16] Ghosh Y.K., Visweswariah S.S., Bhattacharya S. (2000). Nature of linkage between the cationic headgroup and cholesteryl skeleton controls gene transfection efficiency. FEBS Lett.

[17] Kim B.K., Doh K.O., Nam J.H., Kang H., Park J.G., Moon I.J., Seu Y.B. (2009). Synthesis of novel cholesterol-based cationic lipids for gene delivery. Bioorg. Med. Chem. Lett.

[18] Kim B.K., Bae Y.U., Doh K.O., Hwang G.B., Lee S.H., Kang H., Seu Y.B. (2011). The synthesis of cholesterol-based cationic lipids with trimethylamine head and the effect of spacer structures on transfection efficiency. Bioorg. Med. Chem. Lett.

[19] Kim B.K., Doh K.O., Bae Y.U., Seu Y.B. (2011). Synthesis and optimization of cholesterol-based diquaternary ammonium gemini surfactant (chol-gs) as a new gene delivery vector. J. Microbiol. Biotechnol.

[20] Kim B.K., Doh K.O., Hwang G.B., Seu Y.B. (2012). Transfection property of a new cholesterol-based cationic lipid containing tri-2-hydroxyethylamine as gene delivery vehicle. J. Microbiol. Biotechnol.

[21] Tagami T., Barichello J.M., Kikuchi H., Ishida T., Kiwada H. (2007). The gene-silencing effect of sirna in cationic lipoplexes is enhanced by incorporating pdna in the complex. Int. J. Pharm.

[22] Maestrelli F., Gonzalez-Rodriguez M.L., Rabasco A.M., Mura P. (2006). Effect of preparation technique on the properties of liposomes encapsulating ketoprofen-cyclodextrin complexes aimed for transdermal delivery. Int. J. Pharm.

[23] Al-Jamal W.T., Kostarelos K. (2007). Construction of nanoscale multicompartment liposomes for combinatory drug delivery. Int. J. Pharm.

[24] Simberg D., Weiss A., Barenholz Y. (2005). Reversible mode of binding of serum proteins to dotap/cholesterol lipoplexes: A possible explanation for intravenous lipofection efficiency. Hum. Gene Ther.

[25] Yang J.P., Huang L. (1997). Overcoming the inhibitory effect of serum on lipofection by increasing the charge ratio of cationic liposome to DNA. Gene Ther.

[26] Zelphati O., Uyechi L.S., Barron L.G., Szoka F.C. (1998). Effect of serum components on the physico-chemical properties of cationic lipid/oligonucleotide complexes and on their interactions with cells. Biochim. Biophys. Acta.

[27] Audouy S., Molema G., de Leij L., Hoekstra D. (2000). Serum as a modulator of lipoplex-mediated gene transfection: Dependence of amphiphile, cell type and complex stability. J. Gene Med.

[28] Crook K., Stevenson B.J., Dubouchet M., Porteous D.J. (1998). Inclusion of cholesterol in dotap transfection complexes increases the delivery of DNA to cells *in vitro* in the presence of serum. Gene Ther.

[29] Han S.E., Kang H., Shim G.Y., Suh M.S., Kim S.J., Kim J.S., Oh Y.K. (2008). Novel cationic cholesterol derivative-based liposomes for serum-enhanced delivery of sirna. Int. J. Pharm.

[30] Soutschek J., Akinc A., Bramlage B., Charisse K., Constien R., Donoghue M., Elbashir S., Geick A., Hadwiger P., Harborth J. (2004). Therapeutic silencing of an endogenous gene by systemic administration of modified sirnas. Nature.

[31] Ish-I T.I.R., Snip E., Ikeda M., Shinkai S. (2001). [60]fullerene can reinforce the organogel structure of porphyrin-appended cholesterol derivatives: Novel odd-even effect of the (ch2)n spacer on the organogel stability. Langmuir.

[32] Hassani Z., Lemkine G.F., Erbacher P., Palmier K., Alfama G., Giovannangeli C., Behr J.P., Demeneix B.A. (2005). Lipid-mediated sirna delivery down-regulates exogenous gene expression in the mouse brain at picomolar levels. J. Gene Med.

[33] Vijayanathan V., Thomas T., Thomas T.J. (2002). DNA nanoparticles and development of DNA delivery vehicles for gene therapy. Biochemistry.

[34] Manosroi A., Thathang K., Manosroi J., Werner R.G., Schubert R., Peschka-Suss R. (2009). Expression of luciferase plasmid (pcmvluc) entrapped in dppc/cholesterol/ddab liposomes in hela cell lines. J. Liposome Res.

[35] Huang Q.D., Ou W.J., Chen H., Feng Z.H., Wang J.Y., Zhang J., Zhu W., Yu X.Q. (2011). Novel cationic lipids possessing protonated cyclen and imidazolium salt for gene delivery. Eur. J. Pharm. Biopharm.

[36] Biswas J., Bajaj A., Bhattacharya S. (2011). Membranes of cationic gemini lipids based on cholesterol with hydroxyl headgroups and their interactions with DNA and phospholipid. J. Phys. Chem. B.

[37] Islam R.U., Hean J., van Otterlo W.A., de Koning C.B., Arbuthnot P. (2009). Efficient nucleic acid transduction with lipoplexes containing novel piperazine- and polyamine-conjugated cholesterol derivatives. Bioorg. Med. Chem. Lett.

[38] Dabkowska A.P., Barlow D.J., Hughes A.V., Campbell R.A., Quinn P.J., Lawrence M.J. (2012). The effect of neutral helper lipids on the structure of cationic lipid monolayers. J. R. Soc. Interface.

[39] Zhang Y., Bradshaw-Pierce E.L., Delille A., Gustafson D.L., Anchordoquy T.J. (2008). *In vivo* comparative study of lipid/DNA complexes with different *in vitro* serum stability: Effects on biodistribution and tumor accumulation. J. Pharm. Sci.

[40] Duarte S., Faneca H., de Lima M.C. (2011). Non-covalent association of folate to lipoplexes: A promising strategy to improve gene delivery in the presence of serum. J. Control. Release.

[41] Faneca H., Simoes S., de Lima M.C. (2002). Evaluation of lipid-based reagents to mediate intracellular gene delivery. Biochim. Biophys. Acta.

[42] Koster F., Finas D., Schulz C., Hauser C., Diedrich K., Felberbaum R. (2004). Additive effect of steroids and cholesterol on the liposomal transfection of the breast cancer cell line t-47d. Int. J. Mol. Med.

[43] Wheeler C.J., Felgner P.L., Tsai Y.J., Marshall J., Sukhu L., Doh S.G., Hartikka J., Nietupski J., Manthorpe M., Nichols M. (1996). A novel cationic lipid greatly enhances plasmid DNA delivery and expression in mouse lung. Proc. Natl. Acad. Sci. USA.

[44] Ren T., Song Y.K., Zhang G., Liu D. (2000). Structural basis of dotma for its high intravenous transfection activity in mouse. Gene Ther.

[45] Hattori Y., Suzuki S., Kawakami S., Yamashita F., Hashida M. (2005). The role of dioleoylphosphatidylethanolamine (dope) in targeted gene delivery with mannosylated cationic liposomes via intravenous route. J. Control. Release.

[46] Kim W.J., Christensen L.V., Jo S., Yockman J.W., Jeong J.H., Kim Y.H., Kim S.W. (2006). Cholesteryl oligoarginine delivering vascular endothelial growth factor sirna effectively inhibits tumor growth in colon adenocarcinoma. Mol. Ther.

[47] Walczyk D., Bombelli F.B., Monopoli M.P., Lynch I., Dawson K.A. (2010). What the cell “sees” in bionanoscience. J. Am. Chem. Soc.

[48] Capriotti A.L., Caracciolo G., Caruso G., Foglia P., Pozzi D., Samperi R., Lagana A. (2011). Differential analysis of “protein corona” profile adsorbed onto different nonviral gene delivery systems. Anal. Biochem.

[49] Caracciolo G., Pozzi D., Capriotti A.L., Cavaliere C., Foglia P., Amenitsch H., Lagana A. (2011). Evolution of the protein corona of lipid gene vectors as a function of plasma concentration. Langmuir.

[50] Wong A.W., Scales S.J., Reilly D.E. (2007). DNA internalized via caveolae requires microtubule-dependent, rab7-independent transport to the late endocytic pathway for delivery to the nucleus. J. Biol. Chem.

[51] Conner S.D., Schmid S.L. (2003). Regulated portals of entry into the cell. Nature.

[52] Cardarelli F., Pozzi D., Bifone A., Marchini C., Caracciolo G. (2012). Cholesterol-dependent macropinocytosis and endosomal escape control the transfection efficiency of lipoplexes in cho living cells. Mol. Pharm.

[53] Zhang X.X., Allen P.G., Grinstaff M. (2011). Macropinocytosis is the major pathway responsible for DNA transfection in cho cells by a charge-reversal amphiphile. Mol. Pharm.

[54] Bae Y.U., Kim B.K., Park J.W., Seu Y.B., Doh K.O. (2012). Endocytic pathway and resistance to cholesterol depletion of cholesterol derived cationic lipids for gene delivery. Mol. Pharm.

