# Supplementary figures and images for: Efficient Delivery of Plasmid DNA Using Cholesterol-Based Cationic Lipids Containing Polyamines and Ether Linkages
Source: Int J Mol Sci. 2014 Apr 28;15(5):7293–312. doi: 10.3390/ijms15057293 (PMC4057673; doi:10.3390/ijms15057293)

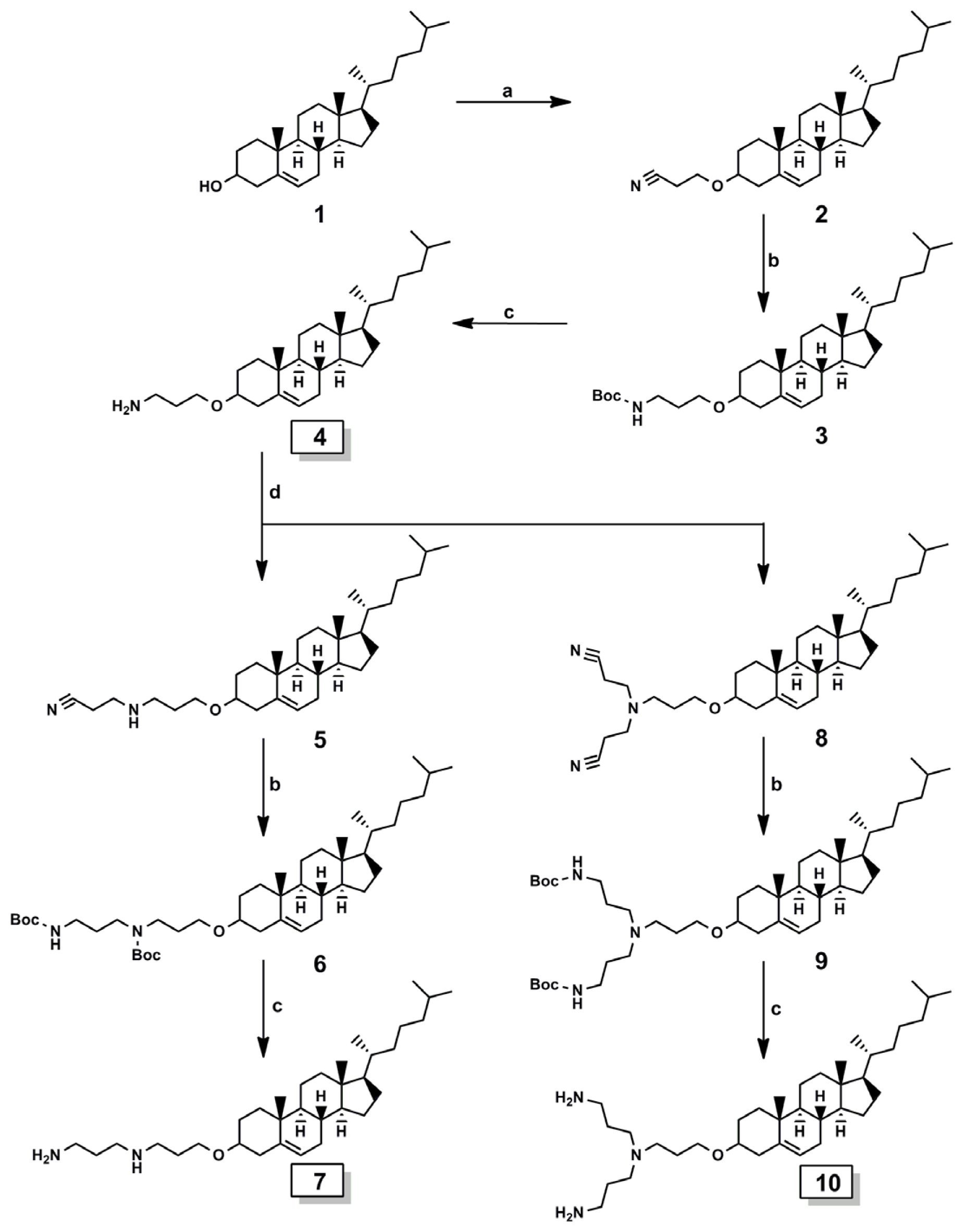

Supplement: Scheme S1. — Synthesis of new cationic lipids. (a) Acrylonitrile, 18-crown-6, aqueous KOH/CH2Cl2 (yield: 100%); (b) NiCl2·6H2O, Boc2O, NaBH4/MeOH (yield: 3, 93%; 6, 80%; 9, 68%); (c) TFA/CH2Cl2 (yield: 4, 85%; 7, 65%; 10, 67%); (d) Acrylonitrile/MeOH, reflux (yield: 5, 42%; 8, 51%; overall, 93%). [file ijms-15-07293s1.tif]

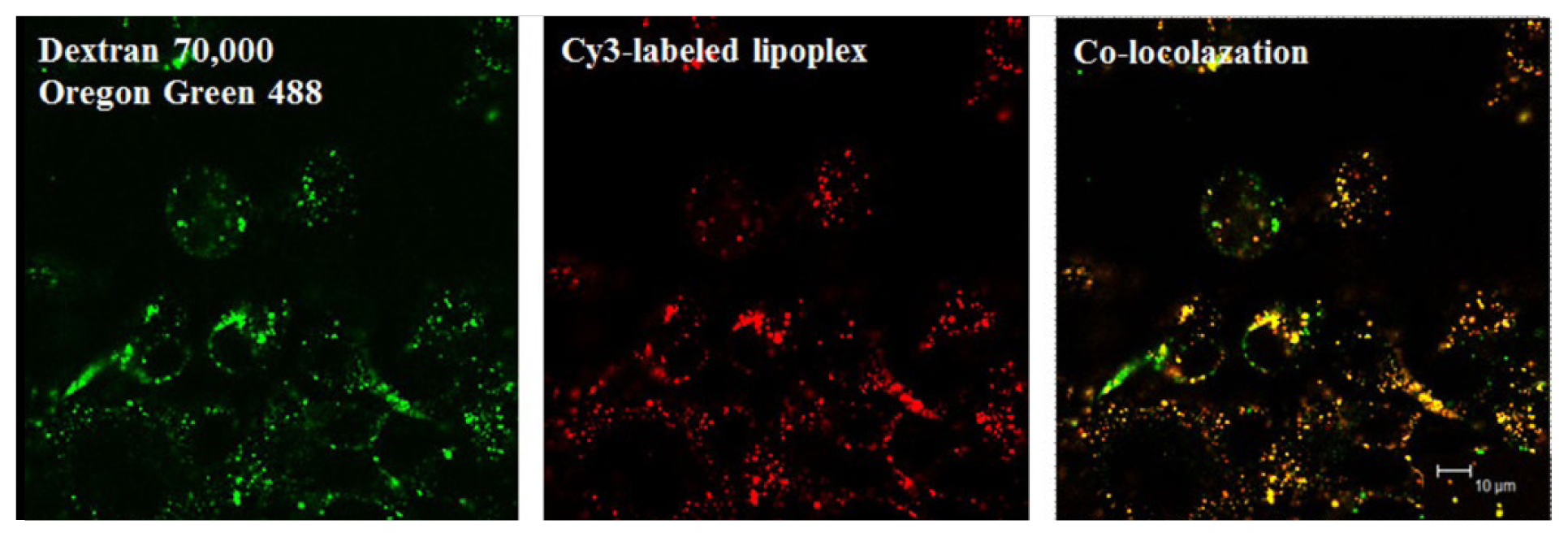

Supplement: Figure S1. — Cellular uptake of the liposome B/DNA complex. We examined the colocalization of lipoplexes with Oregon Green 488-labeled dextran 70,000 molecular weight (dextran 70,000), a known macropinocytic pathway marker. COS-7 cells were incubated with dextran 70,000 (green) and lipoplex B containing Cy3-labeled DNA (red). Images were obtained using confocal microscopy 1 h after transfection. Scale bar: 10 μm. [file ijms-15-07293s2.tif]
